# Changes of Plasma Analytes Reflecting Metabolic Adaptation to the Different Stages of the Lactation Cycle in Healthy Multiparous Holstein Dairy Cows Raised in High-Welfare Conditions

**DOI:** 10.3390/ani11061714

**Published:** 2021-06-08

**Authors:** Michele Premi, Matteo Mezzetti, Giulia Ferronato, Mario Barbato, Fiorenzo Piccioli Cappelli, Andrea Minuti, Erminio Trevisi

**Affiliations:** Department of Animal Sciences, Food and Nutrition (DIANA), Facoltà di Scienze Agrarie, Alimentari e Ambientali, Università Cattolica del Sacro Cuore, 29122 Piacenza, Italy; michele.premi@unicatt.it (M.P.); matteo.mezzetti@unicatt.it (M.M.); giulia.ferronato@unicatt.it (G.F.); mario.barbato@unicatt.it (M.B.); Fiorenzo.piccioli@unicatt.it (F.P.C.); andrea.minuti@unicatt.it (A.M.)

**Keywords:** healthy reference individuals, high-welfare farm, metabolic profile, subclinical disorders

## Abstract

**Simple Summary:**

This study investigates the changes occurring in plasma analytes of healthy multiparous Holstein dairy cows during the dry, the postpartum, the early and the late lactation phases. A welfare assessment at the herd level and a retrospective subclinical diseases screening were used as blocking factors for the selection of reference individuals. Thus, this study provides measurements of the physiological variations affecting plasma analytes concentrations during the pivotal stages of the lactation cycle in a healthy, high welfare-raised subset of reference individuals and suggest an explanation for the underlying processes involved. Finally, we propose reference intervals for plasma analytes in the stages investigated.

**Abstract:**

Here, we tested the changes occurring in several plasma analytes during different stages of the lactation cycle of high welfare raised multiparous Holstein cows, and provided reference intervals (RI) for plasma analytes concentrations. Eleven high-welfare farms (HWF) located in Northern Italy were selected and their herds used to recruit 361 clinically healthy cows undergoing the dry (from −30 to −10 days from real calving; DFC), the postpartum (from 3 to 7 DFC), the early lactation (from 28 to 45 DFC) and the late lactation phases (from 160 to 305 DFC). Cows affected by subclinical diseases (SCD) were retrospectively excluded, and a subset of 285 cows was selected. Data of plasma analytes underwent ANOVA testing using physiological phases as predictors. The individual effect of each phase was assessed using a pairwise *t*-test assuming *p* ≤ 0.05 as a significance limit. A bootstrap approach was used to define the reference interval (RI) for each blood analyte within physiological phases having a pairwise *t*-test *p* ≤ 0.05. The concentration of nonesterified fatty acids, albumin, cholesterol, retinol, paraoxonase and tocopherol changed throughout all the physiological phases, whereas the concentration of K, alkaline phosphatase and thiol groups remained stable. Triglycerides, Zn, and ferric ion reducing antioxidant power in the dry phase and BHB, Ca, myeloperoxidase, haptoglobin, reactive oxygen metabolites and advanced oxidation of protein product in postpartum differed compared with other physiological phases. During the dry phase, Packed cell volume, Cl, and urea concentrations were similar to during the postpartum phase. Similarly, Na, γ-glutamyl transferase and β-carotene concentrations were similar to during the early lactation phase; fructosamine and bilirubin concentrations were similar to during the late lactation phase. During the postpartum phase, fructosamine and P concentrations were similar to during the early lactation phase, and the aspartate transaminase concentration was similar to during the late lactation phase. During the early lactation phase, Mg, creatinine, total protein, globulin and ceruloplasmin concentrations were similar to during the postpartum phase, while the urea concentration was similar to during the late lactation phase. All these plasma analytes differed among the other phases. This study identifies physiological trends affecting plasma analytes concentrations during the different stages of the lactation cycle and provides a guideline for the duration and magnitude of their changes when animals are healthy and raised in optimal welfare conditions.

## 1. Introduction

Dairy cows undergoing different phases of the productive cycle face feed, management and environmental changes, as well as alteration in the endocrine, immune and metabolic assets. These changes are reflected by typical trends of plasma analytes. Thus, assessing multiple plasma analytes at the herd level has the potential to provide an overview on the feeding and management strategies adopted in dairy farms, allowing the prompt evaluation of animal responses to the different phases of the lactation cycle [1]. Trends of plasma analytes included in the metabolic profile of dairy cows have already been investigated during the dry period (from 60 to 10 days before expected calving date; [2,3,4]), the transition to calving (from 7 days before to 7 days after calving [3,5]), the early lactation (from 10 to 100 DIM; [2,5,6,7]) and the mid-late lactation period (from 90 to 215 DIM; [6]). However, an assessment of the welfare status combined with a subclinical disorders (SCD) screening has never been performed on the animals prior their enrollment in the experiment, despite the known role of these factors in affecting the physiological trends of plasma analytes during the lactation cycle [8,9,10].

Stressful conditions due to suboptimal welfare are known to induce severe alteration in plasma analytes, both in humans and dairy cows [2,11,12]. Average welfare conditions in dairy herds could be assessed with several models [13,14,15,16]. Typically, the available models compare specific management strategies adopted in dairy farms (i.e., facilities, environmental condition, feeding strategies) to guidelines suggested by the current legislation, and they include multiple indicators reflecting the relative animal responses to the management strategies (i.e., health status of the herd, reproductive efficiency, animal behavior and performances).

SCDs commonly affect dairy cows in specific phases of their production cycle [17,18]. Although not inducing any evident clinical sings, the occurrence of a SCD is often paired with severe alterations of several plasma analytes [7,19]. Limiting the concept of “healthy cows” to animals showing no clinical diseases could thus result in the inaccurate prediction of the physiological conditions of a population. This is especially true for dairy cows at their transition to calving, when the incidence of SCDs is known to be the highest across the whole lactation cycle [20].

With this work we aim to identify the physiological processes affecting plasma analytes concentrations during the different stages of the lactation cycle and to provide a guideline for the duration and magnitude of their changes when animals are healthy and raised in optimal field conditions. Hence, we tested changes occurring in several plasma analytes concentrations during different stages of the lactation cycle in high welfare raised multiparous Holstein cows, excluding the SCDs. Additionally, we adopted a 95% confidence interval to define reference intervals (RI) for plasma analytes.

## 2. Materials and Methods

The experiment was performed between September 2017 and September 2019, in accordance with Italian laws on animal experimentation (D. Lgs. n. 26, 4 March 2014) and ethics. In the present study, plasma samples previously collected for other studies were used (authorization of the Italian Ministry of Health N 451-2017-PR, N 403-2017-PR, N 484-2018-PR, N 851-2018-PR, N 511-2018-PR, N 296-2019-PR, N 464-2019-PR and N 510-2019-PR). Thirty commercial farms located in the provinces of Brescia, Cremona, Mantova and Piacenza (Northern Italy) were enrolled in this study. The weather in the studied areas was a relatively cool, mid-latitude version of the Humid subtropical climate. The chosen farms raised Italian Holstein dairy cows in freestall barns, adopting a total mixed ration feeding system. 

### 2.1. Welfare Status Evaluation and Criteria for Farm Inclusion

**Figure 1:**
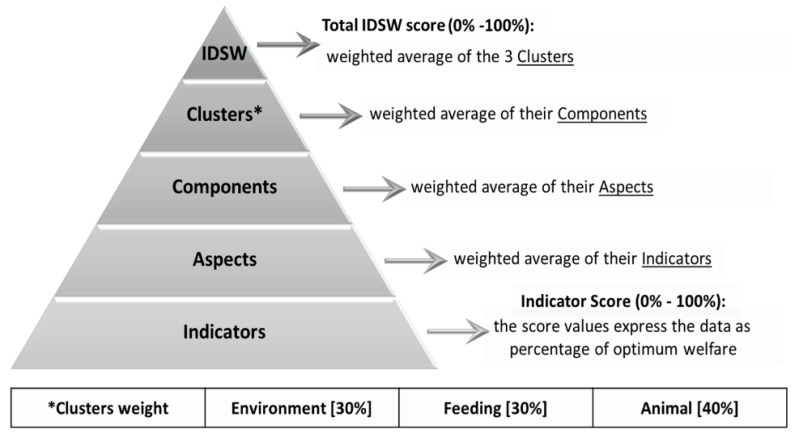
Hierarchical structure and scoring system adopted in the Integrated Diagnostic System Welfare (IDSW) model.

The welfare status of the herds was evaluated by skilled personnel according to the computerized Integrated Diagnostic System Welfare (IDSW) model [13]. The IDSW evaluates the welfare condition of the herd adopting a hierarchical structure (Figure 1), and its outcome has been previously submitted to preliminary validation with blood analytes [21]. Scores are expressed as percentages with 0 and 100% representing the worst and best welfare condition (defined according to well established standards), respectively. The total IDSW score is a pooled-mean of the scores attributed to three “clusters”. The contribution to the total IDSW score is: 30% from the “environment” cluster, 30% from the “feeding” cluster and 40% from the “animal” cluster. Each “cluster” is scored based on the evaluation of several “components”, and each “component” is scored based on the evaluation of several “aspects”. Finally, each aspect is scored based on the evaluation of several specific “indicators”. Detailed description of the sets of “components”, “aspects” and “indicators” considered in the evaluation of each “cluster”, as well as their contribution to the total IDSW score have been defined previously [13].

Dairy farms having a total IDSW score >70% and a score >65% for each cluster were considered high-welfare farms (HWF). Eleven conventional HWF ascribed to the Grana Padano Protected Designation of Origin were selected (number of lactating cows: 322.3 ± 201; milking system: 10 milking parlors; 1 automatic units; milking frequency: 2.1 ± 0.3; days open: 115.5 ± 13.8; energy corrected milk: 10,560 ± 8.1 kg per 305 days lactation), while other farms were discharged. Average technical characteristics, cluster scores and overall IDSW scores assigned to the 11 HWF are available in Table 1. Diet and feed provided in the HWF during the dry, the postpartum, the early lactation and the late lactation phases are presented in Tables S1–S3, respectively.

### 2.2. Criteria for Animal Recruitment

Four physiological phases were defined according to the stage of lactation of the animals (expressed as days from real calving date; DFC): the dry (from −30 to −10 DFC and from 30 to 50 days from dry phase onset), the postpartum (from 3 to 7 DFC), the early lactation (from 28 to 45 DFC), and the late lactation phase (from 160 to 305 DFC). For each HWF, blood samples were collected in a single visit, considering animals undergoing the aforementioned physiological phases at that time. Cows were recruited for blood collection from November until March, to avoid any alteration of the animal physiology due to heat stress. Only cows having parity order ≥2 and ≤5 were considered for blood collection. Prior to the visit by skilled personnel for blood collection, the health status of the herd was checked by veterinary inspection to exclude any cow affected by clinical diseases. On the day of blood collection, the health status of the cows was checked again by skilled personnel. Rectal temperature was measured with a commercial thermometer (Eco-Fast Digital Thermometer, Agri-Pro Enterprises, Iowa Falls, IA, USA), considering fever rectal temperature values >39.5 °C [22,23]. The animals underwent visual inspection for signals indicating a suboptimal physiological status (e.g., abscesses, trauma, mucopurulent or hemorrhagic nasal discharge, eye discharge, hollow left flank caused by an empty rumen and hoof lesions). For the early and the late lactation phases, the individual milk yield of each cow was recorded during the morning milking and compared to the daily milk yield records from the previous week. Animals showing a drop in milk production >15% between the two records were considered at risk of developing a disease [24]. Additionally, the body condition score (BCS) was evaluated in accordance with the Agricultural Development and Advisory Service (1986) [25]. The BCS value was judged as scarce, optimal or excessive, depending on the physiological phase of the animal [26]. According to these criteria, a minimum of eight cows undergoing each physiological phase were selected for blood collection. Animals having fever, signals indicating a suboptimal physiological status, drop of milk yield or BCS values outside the optimal range were not considered for blood collection. For the late lactation phase, animals that were not pregnant were not considered for blood collection. Based on these criteria, 361 clinically healthy multiparous cows, each in different stages of lactation, were enrolled in the experiment (Table 1).

### 2.3. Blood Sample Collection and Analyses

Blood was harvested by means of jugular venipuncture before the morning feeding. Samples were collected in evacuated heparinized tubes (BD Vacutainer, Becton, Dickinson and Co., Franklin Lakes, NJ, USA) and processed as described by Calamari et al. (2016) [27]. After collection, blood was centrifuged (3500× *g*, 15 min at 4 °C) and the Packed cell volume (PCV) was measured through capillary column (ALC Centrifugette 4203) directly on fresh blood after centrifugation. A clinical autoanalyzer (ILAB-650, Instrumentation Laboratory, Bedford, MA, USA) was used to determine the concentration of glucose, nonesterified fatty acids (NEFA), BHB, urea, creatinine, Ca, P, Mg, Na, K, Cl, Zn, aspartate amino transferase–glutamate oxaloacetate transaminase (AST-GOT), gamma glutamyl transferase (GGT), alkaline phosphatase, total protein, haptoglobin, ceruloplasmin, albumin, total bilirubin, cholesterol and globulin in accordance with Calamari et al. (2016) [27]. The reactive oxygen metabolites (ROMt) and ferric ion reducing antioxidant power were determined according to Jacometo et al. (2015) [28], the paraoxonase according to Bionaz et al. (2007) [29], the thiol groups according to Minuti et al. (2014) [30], the myeloperoxidase according to Bradley et al. (1982) [31], the triglycerides according to Bertoni et al. (2008) [32], the fructosamine according to Caré et al. (2018) [33] and the advanced oxidation protein products according to Hanasand et al. (2012) [34]. Retinol, tocopherol and beta-carotene were analyzed by reverse-phase high-performance liquid chromatography (LC-4000, Jasco Europe SRL, Cremella, Italy), as described by Jahan et al. (2015) [35]. Further details on the analytical procedures adopted in blood analysis are reported in Table S4.

### 2.4. Criteria for Retrospective Exclusion, Statistical Analysis and Reference Intervals Calculation

Statistical analyses were performed using R v3.6.1 [36]. Plasma analytes having values outside 1.5 times the interquartile range (above the third quartile or below the first quartile) were defined as outliers and removed from the statistical analysis, in accordance with the American Society for Veterinary Clinical Pathology (ASVCP) guidelines [37]. In all the phases of the lactation cycle, plasma concentrations of BHB > 1.2 mmol/L and Ca < 2.0 mmol/L were assumed as hyperketonemia and hypocalcemia threshold, respectively [38,39]. Animals affected by hyperketonemia or hypocalcemia were retrospectively excluded from the statistical analysis. Furthermore, as other SCDs might not course with changes in plasma biomarkers mentioned above, animals having more than three outlier plasma analytes were retrospectively excluded from the statistical analysis to minimize the risk of including animals affected by unspecific SCDs (i.e., subclinical displaced abomasum, mild mastitis, and severe inflammatory conditions). A total of 76 animals (16 in dry; 22 in postpartum; 21 in early lactation; 17 in late lactation phase) were retrospectively excluded due to SCDs, while plasma samples from 285 healthy Italian Holstein multiparous cows (76 in dry; 81 in postpartum; 63 in early lactation and 65 in late lactation phase) were included in the final database for the statistical analysis.

Before statistical analysis, the normality of distributions was verified for each analyte, assessing skewness and kurtosis using the ‘Skew’ and ‘Kurt’ functions [40], and assuming a ±1.5 range as skewness and kurtosis limits for normal distribution. Analytes showing deviation from normality were transformed through natural logarithms (BHB, AOPP and Zn). Further details on the distribution of blood analytes, and on the number of samples considered for each phase of the lactating cycle, are reported in Table 2.

In order to understand the association of lactation phases on the variability of plasma analytes, data of plasma analytes underwent ANOVA testing using physiological phases as predictors. The statistical model included the fixed effect of the physiological phase (PHASE) for the dry, the postpartum, the early lactation and the late lactation phases. The individual effect of each phase was assessed using a pairwise *t*-test assuming *p* ≤ 0.05 as a significance limit. A bootstrap approach, implemented following Dimauro et al. (2009) [41], and a 95 % confidence interval was used to define the RI for each blood analyte within physiological phases. For each blood analyte, physiological phases having a pairwise *t*-test *p* > 0.05 were merged in RI calculation.

## 3. Results and Discussion

### 3.1. Effect of the Phase of the Lactation Cycle on the Plasma Analytes Concentrations

Values of plasma analytes during different phases of the lactation cycle are presented in Table 3 as mean ± SD.

#### 3.1.1. Packed Cell Volume

The PCV was higher in the dry and the postpartum than in the late lactation phase, and it was higher in the late lactation than in the early lactation phase (*p* < 0.01). Higher PCV was detected in those phases when milk production was suspended or when dairy cows were at the onset of lactation. This observation, paired with the recovery of PCV we detected once the peak of lactation was surpassed, suggests a modification of plasma volume driven by milk production [42,43].

#### 3.1.2. Energy Metabolism Biomarkers

We recorded the lowest concentration of glucose in the postpartum, and it was lower in the early lactation and the dry than in the late lactation phase (*p* < 0.01). The concentration of fructosamine was lower in postpartum and early lactation than in the late lactation and the dry phases (*p* < 0.05 or less). The concentration of NEFA was the highest in the postpartum phase; it was higher in the early lactation than in the dry phase, and higher in the dry than in the late lactation phase (*p* < 0.01). The highest concentration of triglycerides and BHB was recorded in the dry and the postpartum phase, respectively (*p* < 0.01).

Glucose is the primary energy source for metabolic processes, and fasting plasma glucose concentration is finely reflected by fructosamine within one to three weeks [33,44,45]. Plasma NEFA concentration is proportional to the severity of body fat mobilization; triglycerides reflect the ability of the liver in re-esterifying NEFA and exporting them through very low-density lipoproteins (VLDL), while BHB is released in the bloodstream when a NEFA overload impairs the β-oxidation process at the liver level [38,46,47]. We detected the lowest values of glucose and fructosamine concentration within 3 and 7 DFC, consistently with the hypoglycemic state affecting postparturient cows [48]. This is mainly driven by the synthesis of lactose: glucose uptake by the mammary gland for this purpose is known to more than double during the 2 d before parturition, and an even more substantial increase occurs after parturition [49]. Besides mammary gland uptake, immune cells contribute to reducing circulating glucose concentrations in early lactation [50]. Those two metabolic functions drive the glucose availability of early lactating cows, as they are prioritized by the insulin resistance induced in peripheral tissues by growth hormone trends, and by the activation of leukocytes to cope with calving-related insults [51]. Furthermore, the peak of NEFA and BHB concentration we detected in the postpartum is consistent with the mobilization of body fats occurring in this phase [52,53]. In fact, early lactating cows experience negative energy balance (NEB) due to their limited feed intake and their huge glucose requirements, resulting in the mobilization of body reserves to cover this gap [8,46]. The increase of glucose and the reduction of NEFA concentrations we observed in the early lactation compared to the postpartum phase suggest a partial recovery of the homeostasis condition. Nonetheless, the lower glucose and fructosamine, paired with the higher NEFA concentration detected in the early compared to the late lactation phase, suggests that dairy cows do not fully overcome the NEB condition during their first month of lactation. Despite slight differences, the low concentration of glucose and the high concentration of NEFA we found in the dry compared to the late lactation phase suggest a light energy deficit condition, triggered by the increase of glucose requirements occurring in the last month of gestation. These are likely driven by the gravid uterus paired with the low dietary glucose availability ensured by the dry ration compared to the late lactation one [49,54]. Despite that, the higher concentration of triglycerides in the dry compared to the postpartum phase reflects a greater capability of the liver in re-esterifying circulating NEFA before calving, probably due to a higher VLDL availability. This observation is consistent with the rise of plasma concentrations of triglycerides reported in high yielding cows before parturition [55,56].

#### 3.1.3. Protein Metabolism and Kidney Function Biomarkers

The urea concentration was lower in the dry and postpartum phases than in the other physiological phases (*p* < 0.01). Plasma urea concentrations closely reflect the trends of rumen fermentations and could be deeply affected by the availability of different protein sources in the ration. In ruminants, urea is synthetized in the liver from two sources: nitrogen deamination from endogenous amino acids and ammonia absorbed by the rumen [57]. Thus, the trend of plasma urea mostly reflects the low ammonia uptake by the rumen fluid, driven both by the low protein content of the dry rations and the low feed volume contained in the rumen of early lactating cows compared to those in mid and late lactation [58,59]. Furthermore, the substantial differences found in the 11 HWF for the diet crude protein contents (Tables S1–S3) and the likely different length of the fasting conditions of the animals before blood sampling, could have contributed to the wide variability recorded in the plasma urea concentration across the physiological phases considered in this study. Feed analyses aimed at identifying the different protein fractions will likely help understanding the source of such variations in future studies.

The concentration of creatinine was higher in the dry than in the postpartum phase, and it was higher in the postpartum than in the early and late lactation phases (*p* < 0.01). Creatinine is related to the used phosphocreatine during normal muscular activity, but the interpretation of this plasma analyte is challenging. On the one hand, plasma creatinine has been reported to reflect the amount of muscle mass in the body [60,61,62]. On the other hand, the creatinine hematic concentration reflects the kidneys’ ability to remove it, thus serving as a reliable marker of glomerular filtration rate efficiency [63]. The highest concentration of creatinine we detected in the dry phase could thus reflect the greater amount of muscle tissue compared with the lactation phase, consistently with the mobilization of 13 to 25% of body proteins occurring in dairy cows at their transition to calving [62]. Furthermore, creatinine trend could suggest a transient impairment of kidney glomerular filtration rate occurring before calving, similarly to those reported in late-pregnant women [64], and likely driven by body fat mobilization and altered blood pressure during the late gestation phase [65]. Both these interpretations are consistent with the decreased creatinine concentration we detected in the lactating phases, suggesting both a reduced amount of muscle tissue and an improved glomerular filtration rate compared with the dry phase.

#### 3.1.4. Mineral Metabolism Biomarkers

The concentration of Ca was lower in the postpartum than in any other physiological phases (*p* < 0.01). The concentration of P was lower in the postpartum and the early lactation than in the late lactation phase, and it was lower in the late lactation than in the dry phase (*p* < 0.01). The concentration of Mg was lower in the postpartum than in the dry phase, and it was lower in the dry than in the early and late lactation phases (*p* < 0.01). Low Ca and P concentrations detected in the postpartum compared with other phases are consistent with the extremely high demand by the mammary gland at the onset of lactation [66,67]. Ca and P homeostasis are finely regulated by parathormone, which promotes Ca reabsorption from urine and bones and stimulates phosphate absorption at gut level when circulating Ca and P decrease [68,69,70]. Nonetheless, the uptake of circulating Ca and P by the mammary gland at the onset of lactation is too fast to be counterbalanced by these mechanisms, leading to the decrease of their circulating pools [70,71]. Conversely, reduced plasma Mg found in postpartum could be driven by modifications occurring in rumen functions at the onset of lactation [72], as Mg is primely absorbed by rumen epithelium in ruminants.

Among the electrolytes, the concentration of Na was higher in the postpartum than in the dry and the early lactation phases, and it was higher in the dry and the early lactation than in late lactation phase (*p* < 0.01). The concentration of K did not differ between the physiological phases. The concentration of Cl was higher in the dry and the postpartum than in early lactation phase, and it was higher in the early than in the late lactation phase (*p* < 0.01). The higher plasma concentration of Na and Cl detected before calving and at the onset of lactation are consistent with plasma creatinine trends in supporting an impaired glomerular filtration rate occurring in late pregnancy. In fact, an increased plasma electrolyte concentration has been reported in humans affected by kidney dysfunctions, and also in cattle [73,74]. Despite that, increased plasma concentration of electrolytes (hypernatremia) in postpartum cows has also been related to the stressful conditions driven by adrenocorticotropin hormone release and inflammatory conditions occurring around calving [75].

The Zn concentration was higher in the dry phase than in the other physiological phases (*p* < 0.05), as previously observed by Anon (1973) and Pryor (1976) [76,77]. Despite several factors (including inflammatory condition) that are known to contribute to reducing plasma Zn availability in the postpartum phase, a clear explanation behind the reduced concentration of this mineral during lactation phases is still lacking. Moreover, our findings are in contrast with those of previous studies performed on Hereford cattle, reporting the lowest plasma Zn concentration during the dry period and relating these changes to the constriction of the posterior genital tract in late pregnancy [78].

#### 3.1.5. Liver Function Biomarkers

Bilirubin concentration was higher in postpartum than in the early lactation phase, and it was higher in the early lactation than in the dry and the late lactation phases (*p* < 0.01). Among liver damage indicators, the AST-GOT concentration was higher in the postpartum and the late lactation than in the early lactation phase, and it was higher in the early lactation than in the dry phase (*p* < 0.01). The GGT concentration was higher in the late lactation than in the dry and the early lactation phases, and it was higher in the dry and the early lactation than in the postpartum phase (*p* < 0.01). The concentration of ALP did not differ among the physiological phases.

Bilirubin results from degradation of red blood cells. Its plasma concentration could be assumed as a cholestasis index, as it reflects the efficiency of liver enzymes in removing it [79]. Both AST-GOT and GGT are involved in amino acids metabolism, while ALP exerts a role in dephosphorylating compounds. Increased plasma concentration of these enzymes serves as a cytolysis and liver damage biomarker [79]. The higher plasma bilirubin and AST-GOT concentrations found in the postpartum phase suggests transient impairment of liver function, paired with liver cell damages occurring at the onset of lactation. These are probably related to the massive liver activities occurring in this phase, combined with the detrimental effect sorted by mobilized NEFA on the liver functions [80]. The decreased concentration of AST-GOT and bilirubin found in the early lactation phase suggests a gradual recovery of liver function [32]. Both bilirubin and AST-GOT reached their minimum during the dry phase, indicating that this phase displays the best liver status and probably the lower liver activity. Surprisingly, AST-GOT and bilirubin do not overlap with GGT trends in the postpartum phase: the GGT had higher plasma concentrations at the end of lactation and during the dry phase than in early lactation. We hypothesize that this could be due to the concentration of GGT in the plasma fraction at the end of lactation, because the plasma half-life of GGT is most likely longer than that of the AST-GOT [81].

#### 3.1.6. Inflammation and Acute Phase Proteins Biomarkers

The concentration of myeloperoxidase was higher in the postpartum than in the other physiological phases (*p* < 0.01). These trends suggest the activation of leukocytes occurring after calving, as documented by others [82]. In fact, myeloperoxidase is involved in the generation of reactive oxygen species in activated neutrophils, thus serving as a reliable marker of inflammation [83].

Among the positive acute phase proteins (APP), haptoglobin was higher in the postpartum than in the other physiological phases (*p* < 0.01). Ceruloplasmin was higher in the postpartum than in the early and the late lactation phases (*p* < 0.01), and it was higher in the early and late lactation than in the dry phase (*p* < 0.05 or less). Among the negative APP biomarkers, albumin, total cholesterol, and retinol were lower in the postpartum than in the dry phase, in the dry than in the early lactation phase and in the early than in the late lactation phase (*p* < 0.01). The concentration of paraoxonase was lower in the postpartum than in the dry phase, in the dry than in the late lactation phase and in the late lactation than in the early lactation phase (*p* < 0.01). The concentration of total protein and globulin was higher in the early and the late lactation than in the dry phase, and it was higher in the dry than in the postpartum phase (*p* < 0.05 or less).

During an acute phase response, the plasma concentration of positive APP increases as the liver upregulates the synthesis of alpha globulins [84,85]. Conversely, the liver reduces the synthesis of albumin, paraoxonase, retinol-binding protein, and lipoproteins (reflected by plasma concentration of retinol and cholesterol respectively). The drop of their plasma concentration is proportional to the severity of the acute phase response [84,85,86]. Total protein includes serum albumin and globulin; thus, trends of total protein and globulin could serve as reliable markers of changes in plasma protein fractions during the acute phase response. The higher concentration of positive APP, paired with the lower concentration of negative APP and total protein, reflects that a marked acute phase response occurred after calving [32]. The similar trends of myeloperoxidase and acute phase biomarkers also suggest a contribution of activated leukocytes in boosting the severity of the acute phase response in this phase [10]. The reduction of haptoglobin supports the mitigation of the acute phase response condition to occur since the early lactation phase. In fact, haptoglobin is released into the blood at the onset of the acute phase, and its concentration then decreases within 60–96 h [87]. Such a mitigation of the acute phase response in the early lactation phase is further supported by the increase of albumin, total cholesterol, and retinol concentrations. We detected that ceruloplasmin had a delayed recovery of homeostasis condition compared to haptoglobin, as it reached its minimum in the dry phase. Further, ceruloplasmin is known to serve as a longer-lasting plasma biomarker relative to acute phase onset than haptoglobin [88,89].

#### 3.1.7. Oxidant Status Biomarkers

Among the antioxidant systems, tocopherol was lower in the postpartum than in the dry phase, in the dry than in the early lactation phase and in the early lactation than in the late lactation phase (*p* < 0.01). β-carotene was lower in the postpartum than in the early lactation and the dry phases, and it was lower in the early lactation and the dry than in the late lactation phase (*p* < 0.01). Concentration of ferric ion reducing antioxidant power (FRAP) was lowest in the dry phase (*p* < 0.01). Concentration of thiol groups was comparable across physiological phases. Among oxidant species and oxidative stress biomarkers, the highest concentration of ROMt and the lowest concentration of advanced oxidation of protein products (AOPP) were recorded in the postpartum phase (*p* < 0.01).

Tocopherol is known to act as a secondary antioxidant by reducing the chain propagation and amplification of lipid peroxidation. β-carotene is known to exert an indirect antioxidant action by maintaining other antioxidant molecules in their reduced form [90]. FRAP reflects the antioxidant power of bilirubin, uric-acid, proteins and vitamins C and E [91]. Thiols could be used as reliable marker for glutathione availability [92]. ROMt include a wide range of oxidant molecules. AOPP classically reflects protein oxidation driven by hypochlorous acid, thus representing a synthetic marker of oxidative stress caused by activated leukocytes through the respiratory burst [93]. Lower concentrations of tocopherol and β-carotene, paired with higher concentration of ROMt detected in the postpartum phase, are consistent with the depletion of circulating antioxidant systems occurring in early lactating cows as a consequence of the increased oxidative metabolism, mainly driven by the milk synthesis and the activation of the immune system [93,94].

The trends of FRAP and AOPP we detected in postpartum are harder to interpret. We hypothesize that the high FRAP concentration we found in the postpartum compared with the dry phase could be due to the upregulation of body antioxidants synthesis occurring to cope with high ROMt concentrations [92]. This is supported by the high bilirubin concentration found in this phase compared to the dry phase. However, a rise of plasma AOPP was expected in the postpartum phase, when leukocytes were at their maximum activated status. We can speculate that the lower AOPP concentration we found in the postpartum phase could be caused by the primary contribution of albumin in facing oxidative damage, as supported by the detection of nadir for plasma albumin during the postpartum phase. The low ROMt concentration and the high availability of antioxidants (tocopherol and β-carotene) in every other physiological phase compared with the postpartum suggests a recovery of the oxidant/antioxidant balance, likely driven by a mitigation of the oxidative metabolism.

### 3.2. Reference Intervals Calculation

Reference intervals for plasma analytes during the different stages of the lactation cycle are presented in Table 4 as lower limit and upper limit (confidence intervals for 95% observations referred to the reference interval extreme values). We found several differences between the RI defined in this study and those reported in previous works, and the largest discrepancy was detected during the postpartum phase. The minor values of our RI in postpartum were higher for plasma glucose, Ca and P and lower for lipid mobilization-related analytes (NEFA and BHB) than those proposed in previous studies [3,5]. We infer that excluding animals affected by hyperketonemia and hypocalcemia from our reference dataset mitigated the NEB condition and the mineral metabolism dysfunctions faced by the cows at the onset of lactation. Furthermore, the minor values of the RI we identified in postpartum were lower for plasma positive APP (haptoglobin and ceruloplasmin), and higher for plasma albumin and total protein than those proposed by others [3,5]. Such outcomes suggest that the cows in our experiment underwent a milder acute phase response during postpartum phase as compared to previous works [3,5]. Excluding animals having hyperketonemia from our reference dataset could partially account for such a difference, as animals undergoing subclinical ketosis are prone to develop severe acute phase responses at the onset of lactation [19].

Despite positive effects sorted by the rigorous procedures adopted in the recruitment of reference individuals on RIs defined in this study, we caution that the adoption of BHB and Ca as subclinical ketosis and subclinical hypocalcemia markers prevented the exclusion of those animals undergoing other less-specific SCDs (i.e., mild mastitis, subclinical displaced abomasum and mild liver lipidosis). Importantly, the lack of any veterinary inspection concurrent with the blood collection further accrued this limitation in our study, as dedicated veterinary inspections would have likely improved the identification and selection of healthy individuals. Most of the unspecific SCDs are often paired with inflammatory conditions in postpartum cows, leading to the simultaneous variation of multiple plasma analytes concentrations [95]. Consequently, the presence of animals with undetected SCDs within the reference database has the potential to decrease the reliability of RIs. For example, the reduced RI for plasma Ca, P and Zn concentration during the postpartum phase could be expected if animals affected by severe acute-phase responses are included in the reference population. Under this scenario, the conversion of vitamin D to 2,5-hydroxyvitamin D by parathormone is impaired during the acute phase [96], thus accounting for a reduced Ca and P uptake from bone and gut cells in animals having severe acute phase responses after calving, while the liver sequesters circulating Zn from the bloodstream during the acute phase [97]. Further, trends of liver enzymes, liver function indicators and oxidant status biomarkers are deeply affected by the severity of the inflammatory conditions during the postpartum phase [86,94].

Likely, the retrospective exclusion from the reference database of animals with more than three plasma analyte outliers allowed us to remove some of the bias driven by unspecific SCDs from RI calculated in this study, and this could further account for the milder inflammatory state affecting our reference individuals in postpartum as compared with previous studies [3,5]. Despite that, we caution the use of the RIs obtained in this study as “golden standard” for plasma analytes of dairy cows, especially for the postpartum phase. To counteract the possible inclusion in the reference dataset of cows affected by unspecific SCDs, we can speculate that performing a veterinary inspection concurrent with the blood collection and adopting a more stringent confidence interval threshold (with respect to the widely used 95% threshold; [98]) should be considered in future studies to develop reliable RIs for the postpartum phase of dairy cows.

## 4. Conclusions

Our results show that, with the exclusion of K, alkaline phosphatase and thiol groups, trends of all the other investigated plasma analytes differed among the physiological phases considered. We show that assessing welfare condition at the herd level and performing a SCD screening likely reduced the variability among reference individuals used in this study, especially in postpartum phase. The inclusion of BHB as a biomarker for subclinical ketosis accounts for the higher minor value for glucose and the lower minor value for NEFA and BHB, while the inclusion of Ca as a biomarker for subclinical hypocalcemia accounts for the higher minor value for Ca and P we proposed in postpartum as compared with previous works. The inclusion of these specific biomarkers for subclinical disorders and the high welfare standard of the selected herds likely contributed to excluding animals affected by severe acute phase responses in postpartum, as suggested by the lower minor value for haptoglobin and ceruloplasmin, and for the higher minor value for albumin and total protein we proposed as compared with previous works. However, we could not prevent the exclusion of animals affected by unspecific SCDs other than hypocalcemia and hyperketonemia, which likely affected the RIs for postpartum phase. Here, we provided a field-measurement of the physiological variability affecting plasma analytes and a possible explanation for the processes behind the occurrence of such variations in healthy, high-welfare raised multiparous Holstein dairy cows at different stages of the lactation cycle. Although the sets of reference intervals defined in the current study is tightly linked to the population of cows included in the experiment, similar variations of plasma analytes among the four phases of the lactation cycle investigated in this research are likely to be expected in any high-welfare herd.

## Figures and Tables

**Table 1:** Average characteristics, welfare score assigned, and number of cows enrolled for blood collection in the 11 high welfare farms selected for assessing physiological variations of plasma analytes in high welfare raised multiparous Holstein dairy cows during the pivotal stages of the lactation cycle.

Farm	1	2	3	4	5	6	7	8	9	10	11
Average characteristics, unit											
Milking system ^1^	MP	MP	MP	MP	MP	AU	MP	MP	MP	MP	MP
MF ^2^, number	2	2	2	2	2	2.9	2	2	2	2	2
Days open, d	99	109	93	104	119	120	139	118	129	112	128
Lactating cows, number	184	210	394	864	280	107	311	304	175	378	338
ECM ^3^, kg	11,291	10,349	12,435	10,841	11,203	10,055	10,007	10,098	10,025	10,147	9695
Welfare score, %											
Environment cluster	75	75	70	80	70	77	75	66	67	70	66
Feeding cluster	88	87	83	76	87	86	82	83	89	76	84
Animal cluster	79	74	80	74	73	67	71	76	70	72	70
Total IDSW score ^4^	80	78	78	77	76	76	76	75	75	73	73
Cows enrolled, number											
Dry phase	6	6	6	21	10	6	7	6	10	6	8
Postpartum phase	6	6	4	28	10	5	6	6	7	7	18
Early lactation phase	7	6	8	13	10	6	6	2	6	6	14
Late lactation phase	8	6	8	11	8	6	8	6	8	6	7

^1^ MP is milking parlor; AU is automatic unit. ^2^ Daily milking frequency. ^3^ Energy corrected milk for a 305-days lactation: ECM = (milk yield × (0.383 × % fat + 0.242 × % protein + 0.7832)/3.1138). ^4^ Total integrated diagnostic system welfare score = [(Environment cluster score × 0.3) + (Feeding cluster score × 0.3) + (Animal cluster score × 0.4)].

**Table 2:** Samples included in the statistical analysis and skewness and kurtosis values for plasma analytes assessed in healthy, high welfare raised multiparous Holstein dairy cows during the pivotal stages of the lactation cycle.

Item, Unit ^1^	Physiological Phases (Days from Calving)											
	Dry (−30; −10)			Postpartum (+3; +7)			Early Lactation (+28; +45)			Late Lactation (+160; +305)		
	n	Skew ^2^	Kurt ^3^	n	Skew ^2^	Kurt ^3^	n	Skew ^2^	Kurt ^3^	n	Skew ^2^	Kurt ^3^
PCV, L/L	75	0.16	−0.69	78	0.36	−0.39	59	−0.13	−0.68	65	0.12	−0.20
Glucose, mmol/L	74	0.10	−0.26	81	0.18	−0.14	60	0.27	−0.40	64	0.26	−0.19
Fructosamine, mmol/L	75	0.46	−0.18	80	0.38	−0.59	62	0.37	−0.34	65	−0.11	−0.35
NEFA, mmol/L	75	0.93	−0.10	80	1.10	0.92	63	1.17	1.33	64	1.16	1.22
BHB ^‡^, mmol/L	75	0.25	−0.33	80	−0.25	−0.52	63	0.21	0.37	64	0.53	−0.48
Triglycerides, mmol/L	76	0.52	−0.65	75	0.32	−0.36	63	−0.21	−0.21	65	0.06	−0.57
Urea, mmol/L	76	0.31	−0.55	81	0.52	−0.51	63	0.07	−0.49	65	−0.06	−0.41
Creatinine, µmol/L	75	0.29	−0.44	80	0.24	−0.41	63	0.16	−0.45	64	0.06	−0.59
Ca, mmol/L	76	0.30	−0.32	78	−0.08	−0.42	62	−0.05	−0.40	65	−0.33	−0.36
P, mmol/L	65	0.14	−0.20	81	0.45	−0.15	63	−0.42	−0.38	65	−0.09	−0.58
Mg, mmol/L	75	0.19	−0.57	81	0.02	−0.91	63	0.02	−0.91	65	−0.04	−0.68
Na, mmol/L	76	−0.11	−1.09	80	0.05	−0.73	63	0.07	−0.86	65	0.39	−0.69
K, mmol/L	75	0.29	−0.67	79	0.13	−0.26	62	0.07	−0.35	63	−0.26	−0.44
Cl, mmol/L	76	−0.26	−0.73	79	−0.22	−0.89	63	−0.27	−0.38	64	−0.03	−0.68
Zn ^‡^, mmol/L	76	−0.15	−0.05	81	0.18	−0.13	63	−0.10	0.78	62	1.19	3.84
Total bilirubin, µmol/L	73	0.03	−0.82	75	0.52	−0.74	61	0.28	−0.22	65	−0.05	−0.49
AST, U/L	75	0.62	−0.12	72	0.44	−0.30	56	0.16	−0.73	58	0.40	−0.70
GGT, U/L	75	0.57	−0.49	71	0.30	−0.14	57	0.08	0.04	57	0.25	−0.25
ALP, U/L	70	0.18	−0.56	78	0.33	−0.69	62	0.68	−0.21	65	0.36	−0.26
Myeloperoxidase, U/L	73	0.08	−0.42	77	−0.10	−0.44	57	0.37	−0.61	64	−0.10	−0.29
Total protein, g/L	76	−0.18	−0.26	81	0.01	−0.27	63	0.22	−0.63	62	0.09	−0.22
Globulin, g/L	76	0.28	−0.12	80	0.01	−0.27	63	0.40	−0.51	65	0.70	0.10
Haptoglobin, g/L	71	0.56	0.09	81	0.37	−0.71	53	0.20	−0.07	55	0.61	−0.59
Ceruloplasmin, µmol/L	76	0.26	0.09	81	0.16	−0.41	63	0.15	−0.32	62	0.24	−0.21
Albumin, g/L	75	−0.14	−0.68	77	−0.30	−0.15	63	−0.29	−0.15	63	−0.15	−0.38
Cholesterol, mmol/L	69	0.35	−0.08	80	0.20	−0.22	63	0.15	−0.36	61	−0.28	−0.41
Retinol, µmol/L ^4^	73	−0.26	−0.51	81	0.29	−0.73	57	−0.25	−0.54	65	0.32	−0.61
Paraoxonase, U/mL	72	0.48	−0.30	81	0.44	0.03	61	0.10	−0.09	65	0.65	−0.29
Tocopherol, µmol/L	73	0.18	−0.46	80	0.45	−0.40	61	0.39	−0.62	65	0.16	−0.75
β-Carotene, µmol/L	70	0.82	−0.30	69	0.81	0.28	59	0.49	−0.21	59	0.21	−1.01
FRAP, mmol/L	73	0.29	−0.74	78	0.29	−0.97	62	0.58	−0.34	65	−0.47	−0.53
Thiol groups, mmol/L	75	0.40	−0.46	81	0.15	−0.34	62	0.31	−0.50	64	−0.18	−0.84
ROMt, mg H_2_O_2_/dL	75	0.28	−0.26	77	0.03	−0.34	63	−0.01	−0.82	65	−0.05	−0.81
AOPP ^‡^, mmol/L	76	0.23	−1.13	81	0.57	−1.05	62	0.04	−1.65	65	−0.14	−1.11

^1^ PCV = packed cell volume, NEFA = non-esterified fatty acids, BHB = β-Hydroxybutyrate; AST = aspartate aminotransferase; GGT = γ-glutamyl transferase; ALP = alkaline phosphatase; FRAP = ferric reducing antioxidant power, ROMt = total reactive oxygen metabolites; AOPP = advanced oxidation product of protein. ^2^ Skew = Skewness. ^3^ Kurt = Kurtosis. ^4^ Retinol values could be converted into µg/100 mL as follows: µmol/L × 28.645 = µg/100 mL. ^‡^ Variable submitted to logarithmic transformation.

**Table 3:** Coefficient of determination of the statistical model (R^2^) and values of plasma analytes (expressed as mean ± standard deviation) assessed in healthy, high welfare raised multiparous Holstein dairy cows during the different stages of the lactation cycle.

Item ^1^	R^2^	Physiological Phases (Days from Calving)				*p*-Value ^2^					
		Dry (−30; −10)	Postpartum (+3; +7)	Early Lactation (+28; +45)	Late Lactation (+160; +305)	DP × PP	DP × EL	DP × LP	PP × EL	PP × LP	EL × LP
PCV, L/L	0.29	0.34 ± 0.03	0.34 ± 0.02	0.30 ± 0.02	0.33 ± 0.03	ns	***	***	***	***	***
Glucose, mmol/L	0.29	4.22 ± 0.23	3.87 ± 0.34	4.15 ± 0.26	4.35 ± 0.28	***	ns	***	***	***	***
Fructosamine, mmol/L	0.11	288 ± 22.3	273 ± 27.2	278 ± 28.8	298 ± 28.2	***	*	ns	ns	***	***
NEFA, mmol/L	0.55	0.18 ± 0.11	0.67 ± 0.33	0.33 ± 0.19	0.11 ± 0.04	***	***	***	***	***	***
BHB ^‡^, mmol/L	0.26	0.42 ± 0.10	0.67 ± 0.21	0.50 ± 0.19	0.45 ± 0.17	***	ns	ns	***	***	ns
Triglycerides, mmol/L	0.71	0.22 ± 0.05	0.10 ± 0.02	0.10 ± 0.02	0.10 ± 0.02	***	***	***	ns	ns	ns
Urea, mmol/L	0.11	4.22 ± 1.37	4.36 ± 1.20	4.98 ± 1.28	5.22 ± 0.78	ns	***	***	***	***	ns
Creatinine, µmol/L	0.31	97.5 ± 10.5	91.0 ± 8.47	84.3 ± 6.29	84.8 ± 5.50	***	***	***	***	***	ns
Ca, mmol/L	0.23	2.57 ± 0.11	2.40 ± 0.15	2.55 ± 0.13	2.56 ± 0.13	***	ns	ns	***	***	ns
P, mmol/L	0.20	2.02 ± 0.20	1.62 ± 0.45	1.63 ± 0.34	1.86 ± 0.29	***	***	***	ns	***	***
Mg, mmol/L	0.32	0.99 ± 0.08	0.91 ± 0.13	1.09 ± 0.10	1.06 ± 0.10	***	***	***	***	***	ns
Na, mmol/L	0.15	145 ± 4.25	147 ± 3.56	144 ± 4.14	143 ± 3.91	***	ns	***	***	***	***
K, mmol/L	0.01	4.30 ± 0.35	4.23 ± 0.38	4.23 ± 0.48	4.18 ± 0.30	ns	ns	ns	ns	ns	ns
Cl, mmol/L	0.19	106 ± 3.52	106 ± 2.94	104 ± 3.15	103 ± 2.68	ns	***	***	***	***	***
Zn ^‡^, mmol/L	0.04	14.3 ± 2.54	12.6 ± 3.69	13.2 ± 2.71	13.6 ± 3.74	***	*	*	ns	ns	ns
Total bilirubin, µmol/L	0.54	1.67 ± 0.75	5.65 ± 2.79	2.31 ± 1.00	1.54 ± 0.51	***	***	ns	***	***	***
AST, U/L	0.28	83.8 ± 14.9	109 ± 15.5	95.2 ± 12.7	105 ± 21.4	***	***	***	***	ns	***
GGT, U/L	0.34	24.3 ± 6.81	20.5 ± 4.03	25.4 ± 4.52	31.6 ± 6.21	***	ns	***	***	***	***
ALP, U/L	0.04	48.5 ± 13.9	49.2 ± 15.6	42.8 ± 13.6	49.7 ± 12.8	ns	ns	ns	ns	ns	ns
Myeloperoxidase, U/L	0.17	438 ± 54.4	504 ± 53.3	461 ± 55.5	458 ± 61.0	***	ns	ns	***	***	ns
Total protein, g/L	0.41	80.0 ± 5.23	74.0 ± 4.82	82.3 ± 4.35	83.8 ± 3.84	***	***	***	***	***	ns
Globulin, g/L	0.24	43.4 ± 5.51	38.6 ± 4.14	44.6 ± 5.18	45.7 ± 5.01	***	*	***	***	***	ns
Haptoglobin, g/L	0.55	0.17 ± 0.06	0.84 ± 0.51	0.18 ± 0.07	0.13 ± 0.07	***	ns	ns	***	***	ns
Ceruloplasmin, µmol/L	0.25	2.22 ± 0.39	2.93 ± 0.51	2.46 ± 0.56	2.39 ± 0.45	***	***	*	***	***	ns
Albumin, g/L	0.27	36.6 ± 1.60	35.3 ± 2.17	37.6 ± 2.21	38.5 ± 1.75	***	***	***	***	***	***
Cholesterol, mmol/L	0.73	3.05 ± 0.65	2.06 ± 0.46	4.66 ± 1.34	6.13 ± 1.22	***	***	***	***	***	***
Retinol, µmol/L ^3^	0.46	1.04 ± 0.20	0.81 ± 0.27	1.23 ± 0.22	1.41 ± 0.28	***	***	***	***	***	***
Paraoxonase, U/mL	0.27	87.1 ± 16.1	73.2 ± 18.1	101.6 ± 20.3	95.0 ± 16.7	***	***	***	***	***	***
Tocopherol, µmol/L	0.56	5.03 ± 2.03	3.49 ± 1.13	7.26 ± 2.63	10.93 ± 3.80	***	***	***	***	***	***
β-Carotene, µmol/L	0.41	4.02 ± 1.94	2.32 ± 0.98	3.64 ± 1.60	6.74 ± 2.84	***	ns	***	***	***	***
FRAP, mmol/L	0.21	126 ± 13.6	150 ± 25.4	149 ± 21.7	149 ± 18.0	***	***	***	ns	ns	ns
Thiol groups, mmol/L	0.02	338 ± 59.1	351 ± 73.3	354 ± 61.8	361 ± 64.9	ns	ns	ns	ns	ns	ns
ROMt, mg H_2_O_2_/dL	0.18	13.5 ± 3.10	16.6 ± 3.40	13.5 ± 3.63	12.9 ± 2.71	***	ns	ns	***	***	ns
AOPP ^‡^, mmol/L	0.11	63.8 ± 23.3	46.6 ± 18.7	59.0 ± 22.5	63.9 ± 19.0	***	ns	ns	***	***	ns

^1^ PCV = packed cell volume, NEFA = non-esterified fatty acids, BHB = β-Hydroxybutyrate; AST = aspartate aminotransferase; GGT = γ-glutamyl transferase; ALP = alkaline phosphatase; FRAP = ferric reducing antioxidant power, ROMt = total reactive oxygen metabolites; AOPP = advanced oxidation product of protein. ^‡^ Variable submitted to logarithmic transformation. ^2^ DP is dry phase; PP is postpartum phase; EL is early lactation phase; LP is late lactation phase; * is *p* < 0.1; *** is *p* < 0.01. ^3^ Retinol values could be converted into µg/100 mL as follows: µmol/L × 28.645 = µg/100 mL.

**Table 4:** Reference intervals (expressed as lower and upper limits (confidence intervals for 95% observations referred to the reference interval extreme values)) for plasma analytes assessed in healthy, high welfare raised multiparous Holstein dairy cows during the pivotal stages of the lactation cycle.

Item, Unit ^1^	Physiological Phases (Days from Calving)															
	Dry (−30; −10)				Postpartum (+3; +7)				Early Lactation (+28; +45)				Late (+160; +305)			
	Lower Limit		Upper Limit		Lower Limit		Upper Limit		Lower Limit		Upper Limit		Lower Limit		Upper Limit	
PCV, L/L	0.28	(0.28–0.29)	0.39	(0.38–0.40)	0.28	(0.28–0.29)	0.39	(0.38–0.40)	0.25	(0.24–0.26)	0.34	(0.33–0.35)	0.27	(0.26–0.29)	0.37	(0.36–0.39)
Glucose, mmol/L	3.70	(3.63–3.77)	4.68	(4.60–4.75)	3.19	(3.06–3.32)	4.55	(4.43–4.68)	3.70	(3.63–3.77)	4.68	(4.60–4.75)	3.80	(3.69–3.92)	4.90	(4.78–5.02)
Fructosamine, mmol/L	242	(235–250)	343	(336–351)	220	(212–228)	331	(323–339)	220	(212–228)	331	(323–339)	242	(235–250)	343	(336–351)
NEFA, mmol/L	0.00	(0.00–0.01)	0.39	(0.34–0.43)	0.01	(0.00–0.14)	1.33	(1.21–1.46)	0.00	(0.00–0.03)	0.70	(0.62–0.78)	0.02	(0.01–0.04)	0.18	(0.17–0.20)
BHB, mmol/L	0.13	(0.10–0.17)	0.77	(0.74–0.81)	0.26	(0.19–0.34)	1.08	(1.00–1.16)	0.13	(0.10–0.17)	0.77	(0.74–0.81)	0.13	(0.10–0.17)	0.77	(0.74–0.81)
Triglycerides, mmol/L	0.11	(0.09–0.13)	0.32	(0.30–0.34)	0.06	(0.06–0.06)	0.14	(0.13–0.14)	0.06	(0.06–0.06)	0.14	(0.13–0.14)	0.06	(0.06–0.06)	0.14	(0.13–0.14)
Urea, mmol/L	1.70	(1.35–2.05)	6.88	(6.53–7.23)	1.70	(1.35–2.05)	6.88	(6.53–7.23)	3.04	(2.73–3.35)	7.17	(6.86–7.48)	3.04	(2.73–3.35)	7.17	(6.86–7.48)
Creatinine, µmol/L	76.6	(72.5–80.7)	118	(114–122)	74.2	(71.0–77.4)	108	(105–111)	72.9	(71.1–74.7)	96.2	(94.4–97.9)	72.9	(71.1–74.7)	96.2	(94.4–97.9)
Ca, mmol/L	2.32	(2.29–2.35)	2.81	(2.78–2.84)	2.11	(2.05–2.16)	2.7	(2.64–2.76)	2.32	(2.29–2.35)	2.81	(2.78–2.84)	2.32	(2.29–2.35)	2.81	(2.78–2.84)
P, mmol/L	1.62	(1.54–1.70)	2.42	(2.33–2.50)	0.80	(0.69–0.92)	2.44	(2.32–2.55)	0.80	(0.69–0.92)	2.44	(2.32–2.55)	1.30	(1.18–1.42)	2.43	(2.31–2.55)
Mg, mmol/L	0.8	(0.81–0.87)	1.14	(1.11–1.17)	0.67	(0.62–0.71)	1.16	(1.11–1.21)	0.88	(0.85–0.91)	1.27	(1.24–1.30)	0.88	(0.85–0.91)	1.27	(1.24–1.30)
Na, mmol/L	2.32	(2.29–2.35)	2.81	(2.78–2.84)	140	(139–141)	154	(153–156)	2.32	(2.29–2.35)	2.81	(2.78–2.84)	135	(133–136)	150	(149–152)
K, mmol/L	3.47	(3.40–3.55)	5.00	(4.92–5.08)	3.47	(3.40–3.55)	5.00	(4.92–5.08)	3.47	(3.40–3.55)	5.00	(4.92–5.08)	3.47	(3.40–3.55)	5.00	(4.92–5.08)
Cl, mmol/L	99.6	(98.7–101)	113	(112–114)	99.6	(98.7–101)	113	(112–114)	97.7	(96.3–99.0)	110	(109–112)	97.3	(96.2–98.5)	108	(107–109)
Zn, mmol/L	9.26	(8.28–10.3)	19.4	(18.4–20.3)	6.15	(5.34–6.97)	20.0	(19.2–20.8)	6.15	(5.34–6.97)	20.0	(19.2–20.8)	6.15	(5.34–6.97)	20.0	(19.2–20.8)
Total bilirubin, µmol/L	0.33	(0.14–0.51)	2.89	(2.71–3.08)	0.21	(0.00–1.28)	11.1	(10.0–12.2)	0.33	(0.00–0.76)	4.29	(3.86–4.72)	0.33	(0.14–0.51)	2.89	(2.71–3.08)
AST, U/L	54.2	(48.3–60.0)	113	(108–119)	70.8	(65.4–76.2)	143	(138–149)	69.8	(64.1–75.6)	120	(115–126)	70.8	(65.4–76.2)	143	(138–149)
GGT, U/L	12.9	(11.1–14.6)	36.7	(34.9–38.4)	12.5	(10.8–14.1)	28.5	(26.8–30.1)	12.9	(11.1–14.6)	36.7	(34.9–38.4)	19.3	(16.5–22.1)	44.0	(41.2–46.7)
ALP, U/L	19.5	(16.6–22.4)	75.9	(73.0–78.8)	19.5	(16.6–22.4)	75.9	(73.0–78.8)	19.5	(16.6–22.4)	75.9	(73.0–78.8)	19.5	(16.6–22.4)	75.9	(73.0–78.8)
Myeloperoxidase, U/L	336	(322–350)	566	(552–580)	398	(378–419)	609	(589–630)	336	(322–350)	566	(552–580)	336	(322–350)	566	(552–580)
Total protein, g/L	69.5	(67.4–71.6)	90.5	(88.4–92.5)	64.3	(62.5–66.1)	83.6	(81.8–85.4)	74.7	(73.4–75.9)	91.4	(90.1–92.7)	74.7	(73.4–75.9)	91.4	(90.1–92.7)
Globulin, g/L	32.5	(30.4–34.7)	54.4	(52.2–56.5)	30.4	(28.9–32.0)	46.8	(45.3–48.4)	35.1	(33.6–36.6)	55.2	(53.7–56.7)	35.1	(33.6–36.6)	55.2	(53.7–56.7)
Haptoglobin, g/L	0.02	(0.00–0.03)	0.30	(0.28–0.32)	0.00	(0.00–0.02)	1.86	(1.66–2.05)	0.02	(0.00–0.03)	0.30	(0.28–0.32)	0.02	(0.00–0.03)	0.30	(0.28–0.32)
Ceruloplasmin, µmol/L	1.44	(1.29–1.59)	3.00	(2.84–3.15)	1.92	(1.73–2.11)	3.94	(3.75–4.13)	1.42	(1.27–1.58)	3.43	(3.28–3.59)	1.42	(1.27–1.58)	3.43	(3.28–3.59)
Albumin, g/L	33.4	(32.8–34.1)	39.9	(39.2–40.5)	31.0	(30.2–31.9)	39.6	(38.8–40.5)	33.2	(32.3–34.2)	42.0	(41.1–43.0)	35.0	(34.2–35.7)	42.0	(41.2–42.7)
Cholesterol, mmol/L	1.75	(1.49–2.02)	4.34	(4.07–4.60)	1.14	(0.97–1.32)	2.97	(2.80–3.14)	1.98	(1.40–2.56)	7.35	(6.77–7.93)	3.68	(3.15–4.22)	8.57	(8.03–9.10)
Retinol, µmol/L ^2^	0.65	(0.57–0.73)	1.43	(1.36–1.51)	0.29	(0.19–0.39)	1.34	(1.24–1.44)	0.78	(0.68–0.88)	1.67	(1.57–1.77)	0.86	(0.75–0.98)	1.96	(1.84–2.07)
Paraoxonase, U/mL	55.1	(48.6–61.5)	119	(113–126)	37.1	(30.2–43.9)	109	(102–116)	61.7	(52.9–70.4)	142	(133–150)	61.9	(54.9–68.9)	128	(121–135)
Tocopherol, µmol/L	1.04	(0.24–1.84)	9.03	(8.23–9.83)	1.26	(0.83–1.68)	5.72	(5.30–6.15)	2.09	(0.96–3.22)	12.4	(11.3–13.6)	3.38	(1.78–4.98)	18.5	(16.9–20.1)
β-Carotene, µmol/L	0.25	(0.00–0.79)	7.43	(6.89–7.97)	0.38	(0.00–0.77)	4.25	(3.86–4.65)	0.25	(0.00–0.79)	7.43	(6.89–7.97)	1.10	(0.15–2.36)	12.4	(11.1–13.6)
FRAP, mmol/L	98.6	(93.2–104)	153	(147–158)	105	(99.9–110)	193	(188–198)	105	(99.9–110)	193	(188–198)	105	(99.9–110)	193	(188–198)
Thiol groups, mmol/L	221	(208–234)	480	(467–493)	221	(208–234)	480	(467–493)	221	(208–234)	480	(467–493)	221	(208–234)	480	(467–493)
ROMt, mg H_2_O_2_/dL	7.00	(6.25–7.75)	19.6	(18.8–20.3)	9.78	(8.47–11.1)	23.3	(22.0–24.7)	7.00	(6.25–7.75)	19.6	(18.8–20.3)	7.00	(6.25–7.75)	19.6	(18.8–20.3)
AOPP, mmol/L	19.0	(13.8–24.2)	106	(101–111)	9.20	(2.10–16.3)	83.8	(76.8–90.9)	19.0	(13.8–24.2)	106	(101–111)	19.0	(13.8–24.2)	106	(101–111)

^1^ PCV = packed cell volume, NEFA = non-esterified fatty acids, BHB = β-Hydroxybutyrate; AST = aspartate aminotransferase; GGT = γ-glutamyl transferase; ALP = alkaline phosphatase; FRAP = ferric reducing antioxidant power, ROMt = total reactive oxygen metabolites; AOPP = advanced oxidation product of protein. ^2^ Retinol values could be converted into µg/100 mL as follows: µmol/L × 28.645 = µg/100 mL.

## Data Availability

The data presented in this study are available on request from the corresponding author.

## Supplementary Materials

The following are available online at https://www.mdpi.com/article/10.3390/ani11061714/s1, Table S1: Diet used during the dry phase in the 11 high welfare farms included in the study, Table S2: Diet used during the postpartum and early lactation phases in the 11 high welfare farms included in the study, Table S3: Diet used during the late lactation phase in the 11 high welfare farms included in the study, Table S4: Intra- and inter-assay coefficient of variations, limit of quantification (LOQ), codes of commercial kits used, references for their validation in the bovine plasma, calibrators and quality controls used for plasma parameters included in the study. References [99,100,101] are cited in the supplementary materials.
