# Supplementary material for: Changes of Plasma Analytes Reflecting Metabolic Adaptation to the Different Stages of the Lactation Cycle in Healthy Multiparous Holstein Dairy Cows Raised in High-Welfare Conditions
Source: Animals (Basel). 2021 Jun 8;11(6):1714. doi: 10.3390/ani11061714 (PMC8226749; doi:10.3390/ani11061714)
Supplement: Supplementary file 1 [file animals-11-01714-s001.zip › animals-1173080-supplementary.pdf]

animals

MDPI

Supplementary Materials: Changes of Plasma Analytes Reflecting Metabolic Adaptation to the Different Stages of the Lactation Cycle in Healthy Multiparous Holstein Dairy Cows Raised in High-Welfare Conditions

Michele Premi [†], Matteo Mezzetti [†], Giulia Ferronato, Mario Barbato, Fiorenzo Piccioli Cappelli, Andrea Minuti and Erminio Trevisi [*]

Table S1. Diet used during the dry phase in the 11 high welfare farms included in the study.

	**Farm**										
**Item, % dm**	**1**	**2**	**3**	**4**	**5**	**6**	**7**	**8**	**9**	**10**	**11**
Corn silage	58.50	-	-	-	-	52.94	31.49	-	-	33.33	-
Sorghum silage	-	-	-	-	-	-	-	26.61	-	-	-
Ryegrass hay	-	37.50	66.67	50.85	-	-	-	47.15	-	-	81.25
Wheat silage	13.93	-	-	16.95	68.75	-	-	-	68.75	42.00	-
Straw	13.93	37.50	-	12.71	-	29.41	43.49	14.14	-	13.33	-
Corn grain ground	6.69	12.50	-	4.24	6.25	-	2.00	-	6.25	-	-
Soybean meal	5.01	-	-	10.17	6.25	-	10.00	5.53	6.25	-	-
Sunflower meal	-	-	-	-	-	-	2.54	5.78	-	10.67	-
Wheat meal	-	-	-	-	-	-	9.48	-	-	-	-
Molasses beet	-	-		4.24		-	-	-	-	-	-
Supplement	1.95	-	-	0.85	-	-	1.00	0.79	-	-	-
Concentrate [1]	-	12.50	33.33	-	18.75	17.65	-	-	18.75	0.67	18.75
**Nutrient composition, unit [2]**											
ENl, Mcal/kg dm	12.60	9.60	10.79	13.14	12.20	9.20	11.51	12.50	11.70	11.24	11.56
CP, % dm	28.06	21.82	23.60	26.02	27.19	24.87	21.41	19.00	24.25	18.28	22.80
NSC, % dm	49.80	57.05	50.20	49.75	47.11	55.15	58.68	56.00	50.73	57.60	52.20
NDF, % dm	58.50	-	-	-	-	52.94	31.49	-	-	33.33	-

[1] For farms 2, 3, 6 and 10 the analytical components (% dm) of the concentrate were: CP 26,0%, ether extract 1.3%, crude fiber 10.0%, ash 15,4%. For farms 5 and 11 the analytical components (% dm) of the concentrate were: CP 16,8%, ether extract 2.5%, crude fiber 4.5%, ash 18.5%. For farm 9 the analytical components (% dm) of the concentrate were: CP 20.0%, ether extract 4.0%, crude fiber 8.2%, ash 16.4%. [2] ENl is Net energy for lactation; CP is crude protein, NSC is non starch carbohydrates, NDF is neutral detergent fiber.

Table S2. Diet used during the postpartum and early lactation phases in the 11 high welfare farms included in the study.

	Farm										
**Item, % dm**	**1**	**2**	**3**	**4**	**5**	**6**	**7**	**8**	**9**	**10**	**11**
Corn silage	39.82	55.98	48.05	57.27	42.21	55.17	32.94	12.42	56.20	56.51	56.30
Sorghum silage	-	-	-	-	-	-	-	24.60	-	-	-
Wheat silage	16.59	-	-	-	12.99	-	9.49	-	-	-	-
Ryegrass silage	-	5.60	11.44	-	-	-	-	-	-	-	11.02
Alfalfa silage	-	-	-	5.73	-	-	-	-	6.10	-	-
Corn high moisture	8.85	-	-	9.16	13.85	-	-	-	-	-	13.46
Alfalfa hay	7.74	7.63	-	4.58	4.33	6.60	11.48	20.98	3.41	7.37	4.90
Ryegrass hay	-	2.54	4.35	2.29	-	6.60	-	2.12	7.05	6.14	-
Straw	-	-	-	2.29	1.52	-	-	-	-	-	-
Corn grain ground	7.74	10.43	13.27	4.58	7.14	12.30	21.16	12.62	14.09	5.41	4.16
Barley grain ground	-	-	-	-	-	-	-	8.41	-	-	-
Soybean meal	7.96	-	-	6.19	6.06	10.30	10.65	12.10	-	-	6.85
Sunflower meal	-	-	-	-	-	-	4.43	4.34	-	2.95	-
Extruded linseed	1.11		-	-	-	-	-	-	-	-	-
Soybean	-	-	-	2.98	-	2.60	3.32	-	-	-	-
Cotton	-	-	-	-	3.25	-	-	-	-	-	-
Wheat meal	-	-	-	-	-	-	4.32	-	-	-	-
Hydrogenated fat	-	-	-	0.57	-	-	0.52	0.78	-	-	0.73
Molasses beet	-	-	0.23	3.44	2.16	-	-	-	-	-	-
Supplement	-	-	-	0.92	-	-	1.69	1.63	-	1.23	2.6
Concentrate[1]	10.18	17.81	22.65	-	6.50	6.40	-	-	13.18	20.40	-
**Nutrient composition, unit [2]**											
ENl, Mcal/kg dm	1.53	1.64	1.66	1.54	1.61	1.54	1.53	1.55	1.62	1.51	1.62
CP, % dm	16.7	16.2	17.0	15.8	16.4	16.9	16.3	16.5	15.8	16.8	15.1
NSC, % dm	42.0	44.0	37.5	41.3	41.2	39.2	43.1	36.8	40.3	38.0	38.2
NDF, % dm	30.7	31.8	35.6	30.8	31.0	32.4	32.9	35.8	33.8	34.7	36.9

[1] For farm 1 the analytical components (% dm) of the concentrate were: CP 30,0%, ether extract 2.2%, crude fiber 4.5%, ash 6.5%. For farms 2, 6, 9 and 10 the analytical components (% dm) of the concentrate were: CP 31.5%, ether extract 9.8%, crude fiber 11.8%, ash 7.0%. For farm 3 the analytical components (% dm) of the concentrate were: CP 24.0%, ether extract 7.0%, crude fiber 16.0%, ash 9.5%. For farm 5 the analytical components (% dm) of the concentrate were: CP 24.5%, ether extract 10.5%, crude fiber 11.0%, ash 18.0%. [2] ENl is Net energy for lactation; CP is crude protein, NSC is non starch carbohydrates, NDF is neutral detergent fiber.

Table S3. Diet used during the late lactation phase in the 11 high welfare farms included in the study.

	Farm										
**Item, % dm**	**1**	**2**	**3**	**4**	**5**	**6**	**7**	**8**	**9**	**10**	**11**
Corn silage	50.31	55.98	51.68	60.10	42.21	55.17	32.94	12.42	56.20	56.51	56.30
Sorghum silage	-	-	-	-	-	-	-	24.60	-	-	-
Wheat silage	12.58	-	-	-	12.99	-	9.49	-	-	-	-
Ryegrass silage	-	5.60	12.92	-	-	-	-	-	-	-	11.02
Alfalfa silage	-	-	-	6.01	-	-			6.10	-	-
Corn high moisture	10.48	-	-	7.21	13.85	-			-	-	13.46
Alfalfa hay	5.24	7.63	-	6.01	4.33	6.60	11.48	20.98	3.41	7.37	4.90
Ryegrass hay	-	2.54	-	3.61	-	6.60	-	2.12	7.05	6.14	-
Straw	-	-	-	2.88	1.52	-	-	-	-	-	-
Corn grain ground	5.24	10.43	12.92	2.40	7.14	12.30	21.16	12.62	14.09	5.41	4.16
Barley grain ground	-	-	-	-	-	-	-	8.41	-	-	-
Soybean meal	2.10	-	-	6.01	6.06	10.30	10.65	12.10	-	-	6.85
Sunflower meal	-	-	-	-	-	-	4.43	4.34	-	2.95	-
Extruded linseed	1.05		-	-	-	-			-	-	-
Soybean	-	-	-	2.40	-	2.60	3.32	-	-	-	-
Cotton	-	-	-	-	3.25	-	-	-	-	-	-
Wheat meal	-	-	-	-	-	-	4.32	-	-	-	-
Hydrogenated fat	-	-	-	-	-	-	0.52	0.78	-	-	0.73
Molasses beet	-	-	0.26	2.40	2.16	-			-	-	-
Supplement	-	-	-	0.96	-	-	1.69	1.63	-	1.23	2.6
Concentrate[1]	13.00	17.81	22.22	-	6.50	6.40	-	-	13.18	20.40	-
**Nutrient composition, unit** [2]											
ENl, Mcal/kg dm	1.50	1.64	1.59	1,52	1.61	1.54	1.53	1.55	1.62	1.51	1.62
CP, % dm	14.0	16.2	16.3	15.5	16.4	16.9	16.3	16.5	15.8	16.8	15.1
NSC, % dm	42.4	44.0	37.1	43.2	41.2	39.2	43.1	36.8	40.3	38.0	38.2
NDF, % dm	32.8	31.8	36.7	33.6	31.0	32.4	32.9	35.8	33.8	34.7	36.9

[1] For farm 1 the analytical components (% dm) of the concentrate were: CP 30,0%, ether extract 2.2%, crude fiber 4.5%, ash 6.5%. For farms 2, 6, 9 and 10 the analytical components (% dm) of the concentrate were: CP 31.5%, ether extract 9.8%, crude fiber 11.8%, ash 7.0%. For farm 3 the analytical components (% dm) of the concentrate were: CP 24.0%, ether extract 7.0%, crude fiber 16.0%, ash 9.5%. For farm 5 the analytical components (% dm) of the concentrate were: CP 24.5%, ether extract 10.5%, crude fiber 11.0%, ash 18.0%. [2] ENl is Net energy for lactation; CP is crude protein, NSC is non starch carbohydrates, NDF is neutral detergent fiber.

Table S4. Intra- and inter-assay coefficient of variations, limit of quantification (LOQ), codes of commercial kits used, references for their validation in the bovine plasma, calibrators and quality controls used for plasma parameters included in the study.

Parameter, unit [10]	Intra	Inter	LOQ	Kit	Reference	Calibrator	Quality control
Glucose, mmol/L	1.33	0.75	0.1	182508401	-	Homemade bovine standard	Homemade bovine standard; SeraChem Control Level 1, 00181624121; Bov Asy Control 2, AN10263
Fructosamine, mmol/L	0.9	5.33	15	7080	-	Diagnostic Far Calibrator 7080s	Diagnostic Far Control N+P, 7510
NEFA, mmol/L	2.27	5.00	0.01	NEFA-HR(2) R1 Set, 434-917952	-	NEFA standard, 270-77000 [2]	Homemade bovine standard; SeraChem Control Level 1, 00181624121; Bov Asy Control 2, AN10263
BHB, mmol/L	0.82	3.42	0.1	RB10073	-	BHB standard included in the kit	
Triglycerides, mmol/L	0.84	3.44	0.02	18255640	-	Homemade bovine standard	
Urea, mmol/L	1.14	1.23	0.01	182554401	-	Homemade bovine standard	
Ca, mmol/L	0.93	1.86	0.2	182503401	-	Homemade bovine standard	
P, mmol/L	1.64	0.73	0.01	182512401	-	Homemade bovine standard	
Mg, mmol/L	1.00	1.65	0.02	182592401	-	Homemade bovine standard	
Na, mmol/L	0.62	1.53	50	ISE Diluent, 00182534001;	-		
K, mmol/L	0.63	1.11	1.46	ISE Reference, 00182535001	-	ISE low calibrator, 00184692001; ISE high calibrator, 00184693001	Homemade bovine standard; SeraChem Control Level 1, 00181624121; Bov Asy Control 2, AN10263
Cl, mmol/L	0.34	0.77	70	-	-		
Zn, mmol/L	1.76	3.83	0.5	439-149062	-	Bov Asy Control 2 [4]	
Creatinine, µmol/L	1.09	3.09	18	182555401	-	Homemade bovine standard	
Total bilirubin, µmol/L	3.11	2.46	2	182546401	-	Bov Asy Control 2 [4]	
AST, U/L	1.78	3.55	1	182575401	-	No calibration is required	
GGT, U/L	2.27	4.42	2	182576401	-	No calibration is required	
ALP, U/L	0.77	3.52	2	182596401	-	ReferrIL E, 0018256300 [1]	

Myeloperoxidase, U/L	1.83	8.60	10	-	[31]	No calibration is required	Homemade bovine standard; SeraChem Control Level 1, 00181624121
Total protein, g/L	1.07	2.87	10	182514401	-	Homemade bovine standard	
Haptoglobin, g/L	3.64	3.50	0.01	-	[99]	No calibration is required	
Ceruloplasmin, µmol/L	1.39	3.57	0.1	-	[100]	Human plasma ceruloplasmin, A50143H [6]	Homemade bovine standard; SeraChem Control Level 1, 00181624121; Bov Asy Control 2, AN10263
Albumin, g/L	1.20	3.41	16	182500401	-	Homemade bovine standard	
Cholesterol, mmol/L	1.56	1.38	0.1	182505401	-	Homemade bovine standard	
Retinol, µmol/L	2.72	2.70	-	-		R7632	Homemade bovine standard;
Paraoxonase, U/mL	1.38	4.60	1.2	-	[101]	No calibration is required	Homemade bovine standard; SeraChem Control Level 1, 00181624121; Bov Asy Control 2, AN10263
Tocopherol, µmol/L	3.38	3.52	-	-		T3251	Homemade bovine standard;
β-Carotene, µmol/L	5.55	6.32	-	-		C4582	
FRAP, mmol/L	3.13	3.15	30	-	[91]	TROLOX, 238813-1G [8]	Homemade bovine standard; SeraChem Control Level 1, 00181624121; Bov Asy Control 2, AN10263; TROLOX, 238813-1G8
Thiol groups, mmol/L	1.00	5.90	3.5	MC433	-	Calibrator SHp, MC.0309	Homemade bovine standard; SeraChem Control Level 1, 00181624121; Bov Asy Control 2, AN10263 Calibrator SHp, MC.0309

ROMt, mg H2O2/dL	1.40	2.93	3.2	MC0034	-	Calibrator d-ROMs, MC.030 [9]	Homemade bovine standard SeraChem Control Level 1, 00181624121; Control Serum, MC0314; Calibrator d-ROMs, MC0304
AOPP, mmol/L	0.42	10.93	10	-	[34]	Chloramine T trihydrate, 31224-250G [10]	Homemade bovine standard; SeraChem Control Level 1, 00181624121; Bov Asy Control 2, AN10263

[1] Instrumentation Laboratory Werfen, Milano, Italy. [2] Wako Chemicals GmbH, Neuss, Germany. [3] Randox Laboratories Ltd., Crumlin, County Antrim, UK. [4] Diacron International S.r.l., Grosseto, Italy. [5] Megazyme, Wicklow, Ireland. [6] Meridian Life Science, Memphis, TN, USA. [7] Thermo Scientific, Frederick, MD, USA. [8] Sigma-Aldrich Chemie GmbH, Steinheim, Germany. [9] Carlo Erba, Rodano, Milano, Italy. [10] NEFA = non-esterified fatty acids, BHB = β-Hydroxybutyrate; AST = aspartate aminotransferase; GGT = γ-glutamyl transferase; ALP = alkaline phosphatase; FRAP = ferric reducing antioxidant power, ROMt = total reactive oxygen metabolites; AOPP = advanced oxidation product of protein.
